# New Coating Technique of Ceramic Implants with Different Glass Solder Matrices for Improved Osseointegration-Mechanical Investigations

**DOI:** 10.3390/ma6094001

**Published:** 2013-09-11

**Authors:** Enrico Mick, Jana Markhoff, Aurica Mitrovic, Anika Jonitz, Rainer Bader

**Affiliations:** 1 Department of Orthopaedics, Research Lab for Biomechanics and Implant Technology, University Medicine Rostock, Doberaner Strasse 142, Rostock 18057, Germany; E-Mails: jana.markhoff@med.uni-rostock.de (J.M.); anika.jonitz@med.uni-rostock.de (A.J.); rainer.bader@med.uni-rostock.de (R.B.); 2 ZM Praezisionsdentaltechnik GmbH, Breite Strasse 16, Rostock 18055, Germany; E-Mail: info@dcm-management.de

**Keywords:** surface modification, glass solder matrix, ceramic implant, adhesive strength, roughness

## Abstract

Ceramics are a very popular material in dental implant technology due to their tribological properties, their biocompatibility and their esthetic appearance. However, their natural surface structure lacks the ability of proper osseointegration, which constitutes a crucial process for the stability and, thus, the functionality of a bone implant. We investigated the application of a glass solder matrix in three configurations—consisting mainly of SiO_2_, Al_2_O_3_, K_2_O and Na_2_O to TZP-A ceramic specimens. The corresponding adhesive strength and surface roughness of the coatings on ceramic specimens have been analyzed. Thereby, high adhesive strength (70.3 ± 7.9 MPa) was found for the three different coatings. The obtained roughness (R_z_) amounted to 18.24 ± 2.48 µm in average, with significant differences between the glass solder configurations. Furthermore, one configuration was also tested after additional etching which did not lead to significant increase of surface roughness (19.37 ± 1.04 µm) or adhesive strength (57.2 ± 5.8 MPa). In conclusion, coating with glass solder matrix seems to be a promising surface modification technique that may enable direct insertion of ceramic implants in dental and orthopaedic surgery.

## 1. Introduction

Ceramics are frequently used materials in the field of total joint replacement [1]. Especially for bearing surfaces, several mixtures of alumina and zirconia materials have been established due to their excellent tribological properties [2]. Furthermore, the high biocompatibility leads to acceptance by the human body [3]. Therefore, only few and low inflammatory effects by ceramics have been reported [4]. However, this property also involves a severe disadvantage. Due to the minimized interaction with biological tissue, ceramic surfaces do not connect with bone cells properly but rather get encapsulated in fibrous tissue [5,6]. However, present clinical data for zirconia implants is not sufficient to recommend ceramics implants for routine clinical use [7].

Several approaches of surface modifications and coatings for dental and orthopaedic implants have been reported. While some studies focused on covering titanium base bodies with different peptides in order to stimulate bone formation [8,9,10,11], others investigated the effect of calcium phosphate (CaP) or titanium plasma-spray (TPS) coatings [12,13,14,15,16] on implant osseointegration. A further common technique is the treatment of titanium samples by blasting the surface with diverse grits and acid etching, independently or in combination [12,13,14,17,18]. Approaches to coat titanium with colloidal zircon oxide [19] and hardystonite [16] have also been performed.

Surface modifications of zirconia implants become more important as the suitability of ceramic materials in particular for dental applications come to larger awareness. Such approaches mainly include topographical adaptations via sandblasting and/or acid etching [14,20,21,22] or sintering with pore formers [23,24]. However, coating with hydroxyapatite (HA) [23] and calcium liberating titanium oxide (TiO_2_) [25] have also been examined.

Coatings with bioactive glass have already been reported, yet, only on titanium dental implants [15]. The present study investigates modifications of the ceramic surface by means of a glass solder matrix that may allow sufficient osseointegration of ceramic implants in the bone stock or additional mechanically stable coating of the ceramic implants with bioactive or structured layers.

## 2. Materials and Methods

### 2.1. Test Specimens

The ceramic specimens used for present investigation were manufactured by Metoxit AG (Thayngen, Switzerland) according to DIN EN 60267. The TZP-A ceramic (tetragonal zirconia polycrystal with alumina) consists of ZrO_2_, Y_2_O_3_ and Al_2_O_3_ with contents of 95%, 5% and 0.25%, respectively. For mechanical testing discs with 10 mm in diameter and 5 mm in height were fabricated.

### 2.2. Glass Matrix—Mixture, Application and Modification

The examined coatings are glasses of silica based materials taken from the DCMhotbond^®^ series [26]. They can be used for surface conditioning of mixed ceramics or pure zirconia and mainly contain SiO_2_ (60%–70%), Al_2_O_3_ (4%–10%), K_2_O (6%–10%), Na_2_O (6%–10%). Different configurations with varying contents of each component were applied to the TZP-A discs: HT1, LT1 and LT2. While the first is burned at high temperatures, the two other ones are processed at lower temperatures. The layer thickness was exemplarily determined on partly coated specimens using a high resolution (5 µm) caliper gauge (1101-150, INSIZE Co., Ltd., Suzhou New District, China). The corresponding properties, *i.e.*, curing temperature, layer thickness as well as the grit size of the powdery base material are summarized in Table 1.

**Table 1:** Properties and parameters of the investigated glass solder coatings.

Glass solder	Grit size [µm]	Curing temperature [°C]	Layer thickness [µm]
HT1	12.6	1035	30
LT1	24	850	50
LT2	6	800	20

Prior to coating the ceramic specimens are preconditioned via sandblasting (see Table 2) and subsequent evaporation for providing a greaseless surface.

The powdery base material of the glass solder is mixed with an alcoholic fluid. The resulting emulsion of milky appearance is slowly and evenly applied to the ceramic discs using an airbrush operated at 1–1.5 bar and a distance of 10 cm until the surface is entirely covered. The sprayed object is placed on a firing tray and put into an oven (Vario 200, Zubler Geraetebau GmbH, Ulm, Germany) where curing is processed. The temperature rises at a rate of 5–30 K/min up to constant values of 800 up to 1035 °C which are held for 1–3 min, depending on the actual glass solder configuration.

Afterwards, the extremely plain and even glass matrix surface is roughened via sandblasting (see Table 2) and cleaned in an ultrasonic bath with distilled water. For further roughening the specimens coated with HT1 are also treated additionally by resting for 20 min in an acid mixture containing about 7% of 41% hydrofluoric acid and about 10% of 96% sulfuric acid. Then, the samples are neutralized and finally cleaned in an ultrasonic bath with calcium hydroxide solution (lime milk) and distilled water, respectively.

The discs for the mechanical investigations were coated on both end faces.

**Table 2:** Parameters for sandblasting with corundum (Al_2_O_3_, grit size: 110 µm).

Surface type	Jet pressure [bar]	Angle to surface [°]	Distance to surface [cm]
ceramic base body	2	60–80	2–3
glass ceramic coating	1	60–80	2–3

### 2.3. Roughness

Prior to determining the adhesive strength of the coating, the roughness of the surfaces was recorded. For this purpose a profilometer (Hommel-Etamic T1000, Jenoptik AG, Jena, Germany) was used. The surfaces of the coated ceramic discs were tested performing line scans with a tactile length of 8 mm in three different orientations (0°, 60° and 120°). The parameters “mean roughness index” R_a_ and “average surface roughness” R_z_ determined for each of the orientations were averaged giving the value for each specimen represented. Coated/sandblasted (HT1, LT1 and LT2) and coated/sandblasted/etched (HT1) specimens as well as untreated and sandblasted TZP-A specimens and sandblasted titanium specimens were investigated.

An exemplary image of the surface topography after coating with glass solder was created with a scanning electron microscope DSM 960A (Carl Zeiss AG, Oberkochen, Germany).

### 2.4. Adhesive Strength

**Figure 1:**
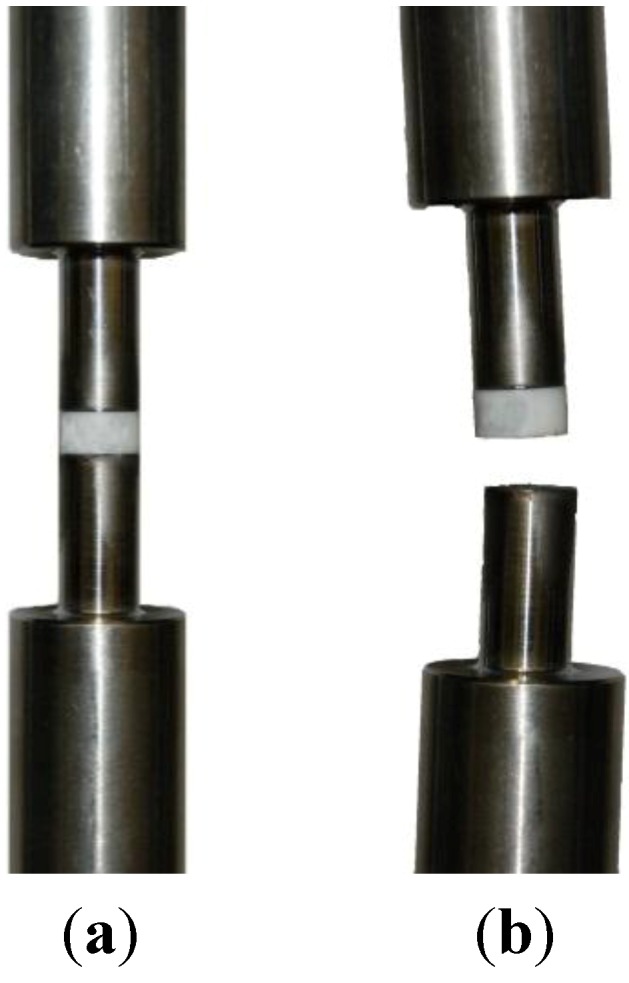
(**a**) Setup for adhesive strength test sample prior to testing; (**b**) Sample after testing.

To evaluate the adhesive strength of the coatings on the ceramic body the TZP-A discs were connected to titanium (Ti6Al4V) cylinders with a diameter of 10 mm (see Figure 1). The cylinders were blasted with Al_2_O_3_ (EK 80) on the end faces. The surfaces were connected using a bonding agent (HTK Ultra Bond 100, HTK Hamburg GmbH, Hamburg, Germany) that cured for 50 min at 180 °C under mechanical pressure. The pull-off test was performed with a universal testing machine (Z050, Zwick GmbH & Co. KG, Ulm, Germany) at a crosshead speed of 5 mm/min. Maximal force was measured and related to the surface area giving the adhesive strength for HT1 (sandblasted and sandblasted/etched), LT1 and LT2 specimens.

### 2.5. X-ray Fluorescence Analysis

In order to make a statement on the qualitative adhesive strength, the titanium end faces needed to be inspected for residues of the surface coating. Therefore, X-ray fluorescence analysis was performed with a Niton^®^ XL3t XRF analyzer (Thermo Fisher Scientific Inc., Munich, Germany). A circular area with a diameter of 8 mm was examined in a single measurement for each specimen. The values given by the analyzer were percentages [%] of the total amount of chemical elements detected on the investigated surface. Several elements that were contained in the coating material at higher ratios had to be ruled out: silicon Si (part of bonding agent), aluminum Al (part of titanium cylinders) and sodium Na (not detectable). In the end, the presence of potassium K on the titanium end face was found to be a suitable decisive criterion as it was contained neither in the titanium cylinders nor in the bonding agent nor in the ceramic base body itself but only in the coating material.

### 2.6. Statistical Analysis

Statistical analysis of results obtained from mechanical testing was performed using SPSS Statistics (v20, IBM Corp., Armonk, NY, USA). A one-way ANOVA and a post-hoc Bonferroni test with a significance level of *p* = 0.05 were conducted.

## 3. Results and Discussion

### 3.1. Roughness

As a reference eight untreated and eight sandblasted TZP-A discs as well as the sandblasted (sb) end faces of eight titanium cylinders were investigated. Table 3 summarizes the corresponding roughness parameters. For untreated TZP-A a mean roughness index of R_a_ = 0.20 ± 0.03 µm and an average surface roughness of R_z_ = 1.57 ± 0.16 µm were determined, proving the smooth character of untreated ceramic surfaces. Sandblasting of the TZP-A specimens lead to roughness values of R_a_ = 0.65 ± 0.08 µm and R_z_ = 4.28 ± 0.61 µm. For titanium, the values were R_a_ = 0.78 ± 0.11 µm and R_z_ = 5.45 ± 0.61 µm, enabling osseointegration to a reasonable extent as shown by other authors before [12,13,18].

**Table 3:** Roughness parameters for reference samples (TZP-A and titanium).

Sample type	No. of samples	Ø R_a_ [µm]	Ø R_z_ [µm]
TZP-A (untreated)	8	0.20 ± 0.02	1.55 ± 0.12
TZP-A (sb)	8	0.65 ± 0.08	4.28 ± 0.61
titanium (sb)	8	0.78 ± 0.11	5.45 ± 0.61

Ceramic specimens coated with HT1 were investigated after sandblasting (sb) and after additional etching (sb/et). The roughness parameters were not influenced connotatively due to the process of etching as shown in Table 4. Neither the differences for R_a_ nor for R_z_ were significant (*p* > 0.05). This might indicate a certain resistance of the glass solder matrix against chemical treatment. Another explanation would be a balance between the roughening effect and the blunting of the topography generated by sandblasting.

**Table 4:** Roughness parameters for modified TZP-A ceramic surfaces.

Sample type	No. of samples	Ø R_a_ [µm]	Ø R_z_ [µm]
HT1 (sb)	6	3.61 ± 0.23	20.44 ± 1.23
HT1 (sb/et)	6	3.31 ± 0.19	19.37 ± 1.04
LT1 (sb)	6	2.90 ± 0.48	17.29 ± 2.80
LT2 (sb)	6	2.83 ± 0.19	16.99 ± 1.35

As described in Section 2.2, etching needs to be followed by neutralization and cleaning. Every work step during the manufacture of an implant bears a risk of failures and mistakes, and especially acid residues on implant surfaces may severely affect the biocompatibility. The etching treatment did not show a positive effect; therefore, the other surface modifications (LT1 and LT2) were only tested in a sandblasted condition. The corresponding roughness parameters are shown in Table 4. For the surface modifications LT1 and LT2, similar roughness values without significant differences were revealed. However, the roughness parameters of HT1 were significantly higher than those of LT1 and LT2. Generally, the roughness values of all glass solder coatings were significantly higher than those of untreated (factor 11 to 13 for R_z_) and sandblasted TZP-A specimens (factor 4.0 to 4.8 for R_z_) and those of sandblasted titanium specimens (factor 3.1 to 3.7 for R_z_).

**Figure 2:**
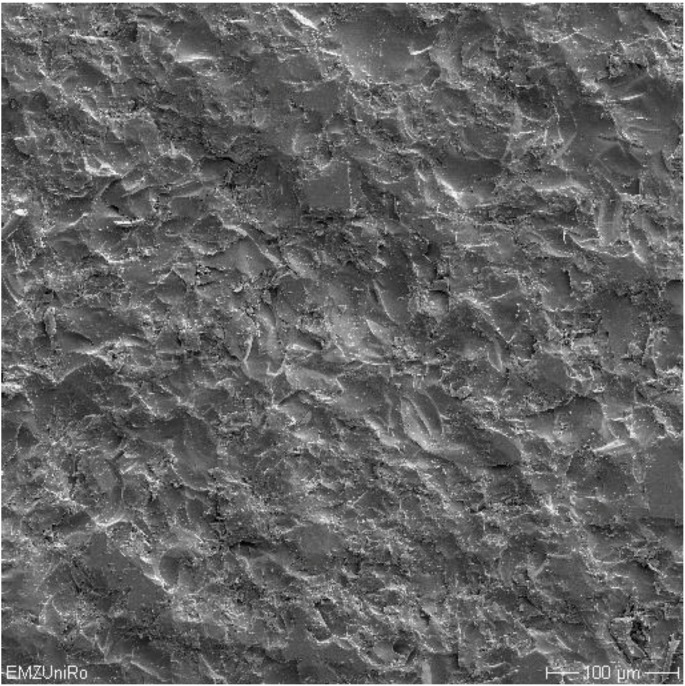
SEM image (magnification: 200X) of a TZP-A specimen coated with glass solder matrix HT1.

**Figure 3:**
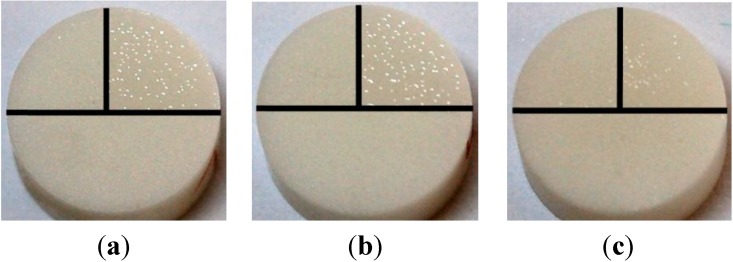
TZP-A specimens partly coated with glass solder: (**a**) HT1; (**b**) LT1; and (**c**) LT2 with each bottom half being uncoated, the right upper quarter being coated with raw glass solder and the left upper quarter being coated and sandblasted.

Figure 2 shows an exemplary scanning electron microscope (SEM) image of the surface topography of a TZP-A specimen coated with HT1 after sandblasting. The translucent appearance of each coating with glass solder matrix is illustrated in Figure 3.

HT1 seems slightly more favorable than the others because it showed the highest average surface roughness as well as the lowest standard deviation with respect to R_z_.

### 3.2. Adhesive Strength and X-ray Fluorescence Analysis

Adhesive strength tests were performed on the surface modifications HT1, LT1 and LT2 which were applied to the TZP-A discs and roughened via sandblasting thereafter. Again, the modification HT1 was also tested in an acid etched condition to investigate a possible influence of etching. The corresponding ultimate forces and stresses as well as the percentages of potassium on the titanium end faces are summarized in Table 5. Potassium and thus coating was released in every case. This is in accordance with the detected adhesive strength values, which are all lower than the tensile strength of the bonding agent (~100 MPa). However, the minimum adhesive strength as demanded by ASTM standard F-1147 (22 MPa) was reached in every case. No significant differences in adhesive strength could be observed (*p* > 0.05). Strength values obtained for HT1 with additional etching (57.2 ± 5.8 MPa) were slightly lower than those of HT1 in sandblasted condition (72.4 ± 11.8 MPa), which were the highest of all variations investigated. LT1 and LT2 showed a steady and in addition a similar behavior with LT1 having a slightly higher strength (71.3 ± 2.1 MPa). On the other hand, LT1 seems to be more brittle as quite large amounts of coating were released from the TZP-A base body (2.3% ± 1.3% *vs.* 0.6% ± 0.2%). However, the highest potassium rates were determined for both HT1 modifications (5.5% ± 2.9% (sb) and 3.9% ± 3.4% (sb/et)). Yet, none of the differences were significant (*p* > 0.05).

**Table 5:** Summary of results from adhesive strength testing and X-ray fluorescence analysis.

Sample type	No. of samples	Ultimate force [N]	Adhesive strength [MPa]	K on Ti face [%]
HT1 (sb)	3	5687 ± 928	72.4 ± 11.8	5.5 ± 2.9
HT1 (sb/et)	3	4491 ± 453	57.2 ± 5.8	3.9 ± 3.4
LT1 (sb)	3	5601 ± 167	71.3 ± 2.1	2.3 ± 1.3
LT2 (sb)	3	5284 ± 424	67.3 ± 5.4	0.6 ± 0.2

Highest values in surface roughness and adhesive strength were found for HT1. In consideration of the fact that all investigated coating configurations showed similar mechanical properties, only tendencies for a most suitable glass solder can be derived.

Despite the promising results the present study has still some limitations. First of all, the processes of applying the coating powder to the ceramic surface as well as all sandblasting operations were performed manually. In order to produce glass solder layers of consistent thickness and quality, the process should be established in an automated production chain. Furthermore, some issues still have to be examined, comprising the optimization of the application process and mechanical and cellbiological tests of the specimens. For instance, four-point-bending on coated ceramic rods has to be performed to determine a potential influence of the surface modification on the bending strength of ceramic base material. Also, cell proliferation on the coated surfaces has to be analyzed. Moreover, *in vivo* experiments investigating the integration of coated ceramic implants into the bone stock are required before clinical application of the new surface coating.

## 4. Conclusions

In the present study the mechanical properties of coatings for ceramic implants revealed promising results testing different glass solder matrix variations. Due to the investigated surface modifications roughness values could be achieved that were significantly higher than those of sandblasted TZP-A and sandblasted titanium which constitutes a kind of gold standard. Furthermore, it was found that all configurations possessed fairly sufficient adhesive strength indicating that the coating can resist the mechanical loads of dental and orthopaedic implants. Another finding was the lack of impact of additional etching on the surface topography and the adhesive strength. Therefore, this process was omitted. The three investigated configurations showed similar properties with the tendency of one variation being most suitable with respect to mechanical testing. In general, the coating with glass solder matrix constitutes an auspicious surface modification technique for enabling direct insertion of ceramic implants in dental and orthopaedic surgery.
